# Improved Pre-miRNAs Identification Through Mutual Information of Pre-miRNA Sequences and Structures

**DOI:** 10.3389/fgene.2019.00119

**Published:** 2019-02-25

**Authors:** Xiangzheng Fu, Wen Zhu, Lijun Cai, Bo Liao, Lihong Peng, Yifan Chen, Jialiang Yang

**Affiliations:** ^1^College of Information Science and Engineering, Hunan University, Changsha, China; ^2^School of Mathematics and Statistics, Hainan Normal University, Haikou, China; ^3^School of Computer Science, Hunan University of Technology, Zhuzhou, China; ^4^Department of Genetics and Genomic Sciences, Icahn Institute for Genomics and Multiscale Biology, Icahn School of Medicine at Mount Sinai, New York, NY, United States

**Keywords:** pre-miRNAs identification, feature representation algorithm, mutual information, structure analysis, support vector machine

## Abstract

Playing critical roles as post-transcriptional regulators, microRNAs (miRNAs) are a family of short non-coding RNAs that are derived from longer transcripts called precursor miRNAs (pre-miRNAs). Experimental methods to identify pre-miRNAs are expensive and time-consuming, which presents the need for computational alternatives. In recent years, the accuracy of computational methods to predict pre-miRNAs has been increasing significantly. However, there are still several drawbacks. First, these methods usually only consider base frequencies or sequence information while ignoring the information between bases. Second, feature extraction methods based on secondary structures usually only consider the global characteristics while ignoring the mutual influence of the local structures. Third, methods integrating high-dimensional feature information is computationally inefficient. In this study, we have proposed a novel mutual information-based feature representation algorithm for pre-miRNA sequences and secondary structures, which is capable of catching the interactions between sequence bases and local features of the RNA secondary structure. In addition, the feature space is smaller than that of most popular methods, which makes our method computationally more efficient than the competitors. Finally, we applied these features to train a support vector machine model to predict pre-miRNAs and compared the results with other popular predictors. As a result, our method outperforms others based on both 5-fold cross-validation and the Jackknife test.

## Introduction

Derived from hairpin precursors (pre-miRNAs), mature microRNAs (miRNAs) belong to a family of non-coding RNAs (ncRNAs) that play significant roles as post-transcriptional regulators (Lei and Sun, 2014). For example, hypothalamic stem cells partially control aging rate through extracellular miRNAs (Zhang et al., 2017). MiRNAs are formed by cleavage of pre-miRNAs by enzymes. Discovery of miRNAs relies on predictive models for characteristic features from pre-miRNAs. However, the short length of miRNA genes and the lack of pronounced sequence features complicate this task (Lopes et al., 2016). In addition, miRNAs are involved in many important biological processes, including plant development, signal transduction, and protein degradation (Zhang et al., 2006; Pritchard et al., 2012). Due to their intimate relevance to miRNA biogenesis and small interfering RNA design, pre-miRNA prediction has recently become a hot topic in miRNA research. However, traditional experimental methods like ChIP-sequencing are expensive and time-consuming (Bentwich, 2005; Li et al., 2013; Liao et al., 2014; Peng et al., 2017). In the post-genome era, a large number of genome sequences have become available, which provides an opportunity for large scale pre-miRNA identification by computational techniques (Li et al., 2010).

In recent years, many computational methods have been proposed to identify pre-miRNAs, most of which are based on machine learning (ML) algorithms or statistical models. The ML-based methods usually model pre-miRNA identification as a binary classification problem to discriminate real and pseudo-pre-miRNAs. Widely used ML-based algorithms include support vector machines (SVMs) (Xue et al., 2005; Helvik et al., 2007; Huang et al., 2007; Wang Y. et al., 2011; Lei and Sun, 2014; Lopes et al., 2014; Wei et al., 2014; Liu et al., 2015b; Khan et al., 2017), back-propagation and self-organizing map (SOM) neural networks (Stegmayer et al., 2016; Zhao et al., 2017), linear genetic programming (Markus and Carsten, 2007), hidden Markov model (Agarwal et al., 2010), random forest (RF) (Jiang et al., 2007; Kandaswamy et al., 2011; Lin et al., 2011), covariant discrimination (Chou and Shen, 2007; Lopes et al., 2014), Naive Bayes (Lopes et al., 2014), and deep learning (Mathelier and Carbone, 2010). For example, Yousef et al. (2006) Peng et al. (2018) used a Bayesian classifier for pre-miRNA recognition, which has demonstrated effectiveness in recognizing pre-miRNAs in the genomes of different species. Xue et al. (2005) proposed a triplet-SVM predictor to identify pre-miRNA hairpin structural features, whose prediction performance has been improved by 10% in a later method using a RF-based MiPred classifier (Jiang et al., 2007). In addition, Stegmayer et al. (2016) proposed a deepSOM predictor to solve the problem of imbalance of positive and negative pre-miRNA samples.

It is known that the performance of ML-based methods is highly associated with the extraction of features (Liao et al., 2015b; Zhang and Wang, 2017; Ren et al., 2018). Typical feature representation methods include secondary structure and sequence information-based methods (Wei et al., 2016; Saçar Demirci and Allmer, 2017; Yousef et al., 2017). For example, Xue et al. (2005) proposed a 32-dimensional feature of triplet sequences containing secondary structure information to better express pre-miRNA sequences. Jiang et al. (2007) performed random sequence rearrangement, which is useful in obtaining the energy characteristics of pre-miRNA sequences. However, this method is quite slow. In addition, Wei et al. (2014) and Chen et al. (2016) extended the features proposed by Xue et al. (2005) into 98-dimensional pre-miRNA features, which resulted in a better pre-miRNA prediction accuracy. Most pre-miRNAs have the characteristic stem–loop hairpin structure (Xue et al., 2005); thus, the secondary structure is an important feature used in computational methods. Recently, Liu et al. proposed several methods for predicting pre-miRNAs on the basis of the secondary structure, namely, iMiRNA-PseDPC (Liu et al., 2016), iMcRNA-PseSSC (Liu et al., 2015b), miRNA-dis (Liu et al., 2015a), and deKmer (Liu et al., 2015c). Some researchers (Khan et al., 2017; Yousef et al., 2017) have increased the dimensionality of features by combining multi-source features to improve the accuracy of pre-miRNAs prediction. With the increase of feature dimension, considerable redundant information and noises are also incorporated, which may reduce the prediction accuracy and slow down the algorithm. Thus, it is usually necessary to perform feature selection to remove irrelevant or redundant features. An excellent feature selection method can effectively reduce the running time for training the model and improve the performance of the prediction (Wang X. et al., 2011; Wang Y. et al., 2011). To further facilitate computational processes, several bioinformatics toolkits have been developed to generate numerical sequence feature information (Liu et al., 2015d).

Developing an effective feature representation algorithm for pre-miRNA sequences is a challenging task. Existing methods have several drawbacks, which may not be sufficiently informative to distinguish between pre-miRNAs and non-pre-miRNAs. First, even excellent feature extraction methods usually only consider the frequency or sequence information of the bases of pre-miRNA sequences while ignore the interaction between two bases. Second, feature extraction methods based on secondary structures usually only consider the global characteristics while ignore the mutual influence of the local characteristics of structures. Third, methods combining multisource feature information and integrating feature selection algorithms to reduce dimensionality (Khan et al., 2017; Yousef et al., 2017) is inefficient in computational time.

As a useful measure to compare profile information based on their entropy, mutual information (MI) has been extensively applied in computational and bioinformatics studies. For instance, MI profiles were used as genomic signatures to reveal phylogenetic relationships between genomic sequences (Bauer et al., 2008), as a metric of phylogenetic profile similarity (Date and Marcotte, 2003), and for predicting drug-target interactions (Ding et al., 2017) and gene essentiality (Nigatu et al., 2017). Inspired by previous studies (Date and Marcotte, 2003; Bauer et al., 2008; Ding et al., 2017; Nigatu et al., 2017; Zhang and Wang, 2018), we proposed a novel MI-based feature representation algorithm for sequences and secondary structures of pre-miRNAs. Specifically, we used entropy and MI to calculate the interdependence between bases, and calculated the 3-gram MI and 2-gram MI of the sequences and secondary structures as feature vectors, respectively. Due to the nature of MI in representing profile dependency, our method is capable of catching the interactions between sequence bases and local features of the secondary structure, which is critical to pre-miRNA prediction. In addition, we combined the MI feature with the minimum free energy (MFE) feature of pre-miRNA, one of the most widely used features for RNA study and constructed a total of 55-dimensional features. Since the feature space is smaller than that of most popular methods, our method is computationally more efficient than the competitors while keeping most important information for pre-miRNA prediction. Our method was evaluated on a stringent benchmark dataset by a jackknife test and compared with a few canonical methods.

## Materials and Methods

### Framework of the Proposed Method

**Figure 1:**
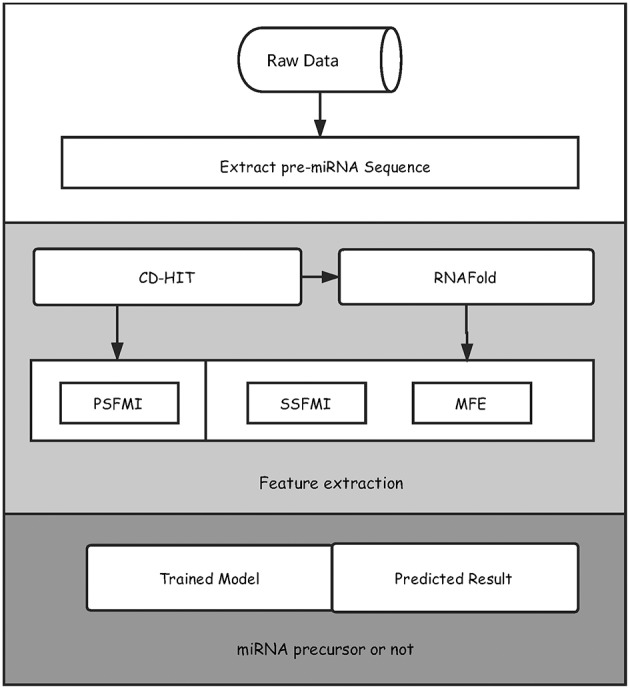
The overall framework of the proposed method for predicting pre-miRNAs.

We illustrated in Figure 1 the overall framework of our method, which consists of two main steps, namely, feature extraction and pre-miRNA prediction. In the feature extraction step, the initial pre-miRNA sequences were first extracted from the raw data. Secondly, homology bias was avoided by using the CD-HIT software (Li and Godzik, 2006) (with threshold value 0.8), and the samples with similarity greater than the threshold in the initial dataset were filtered out. The remaining data was used as the benchmark dataset for this study. After that, the secondary structures of the sequences in the benchmark dataset were predicted by the software RNAfold (Hofacker, 2003). Finally, the primary sequence features based on mutual information (PSFMI), secondary structure features based on mutual information (SSFMI), and MFE features were retrieved, respectively for samples in the benchmark dataset. In the pre-miRNA prediction step, the generated features were fed into an SVM classifier to generate a training model, which was employed to predict pre-miRNAs.

### Datasets

#### Balanced Dataset

Our balanced benchmark dataset for pre-miRNA identification consists of real Homo sapiens pre-miRNAs as positive set and two pseudo pre-miRNAs subsets as negative set, named as: *S*_1_ and *S*_2_, respectively. The benchmark dataset *S*_1_ and *S*_2_ can be formulated as:

$$\begin{array}{l} S_{1}=S^{+}\cup S_{xue(1612)}^{-}\\ S_{2}=S^{+}\cup S_{wei(1612)}^{-} \end{array}$$

The benchmark dataset *S*^+^contains a total of 1,612 positive samples, which were selected from the 1,872 reported Homo sapiens pre-miRNA entries downloaded from the miRBase (20th Edition) (Kozomara and Griffithsjones, 2011), and the pre-miRNAs sharing sequence similarity more than 80% were removed using the CD-HIT software (Li and Godzik, 2006) to get rid of redundancy and avoid bias; the negative samples set $S_{xue}^{-}$ contains 1,612 pseudo miRNAs, which were selected from the 8,494 pre-miRNA-like hairpins $S_{xue}^{-}$ (Xue et al., 2005); the $S_{wei}^{-}$ contains 1,612 pseudo miRNAs, which were selected from the 14,250 pre-miRNA-like hairpins $S_{wei}^{-}$ (Wei et al., 2014).

In addition, we selected 88 new pre-miRNA sequences from a later version (e.g., miRBase22) as positive samples, and selected 88 samples from $S_{wei}^{-}$ as negative samples to construct a benchmark dataset for independent testing, named *S*_3_. The benchmark dataset *S*_3_ can be formulated as:

$$\begin{array}{l} S_{3}=S_{miR22}^{+}\cup S_{wei\left(88\right)}^{-} \end{array}$$

#### Imbalanced Dataset

To evaluate the performance of our approach in an unbalanced dataset, we have constructed two unbalanced benchmark datasets, named as: *S*_4_ and *S*_5_, respectively. The benchmark dataset *S*_4_ and *S*_5_ can be formulated as:

$$\begin{array}{l} S_{4}=S^{+}\cup S_{wei}^{-}\\ S_{5}=S_{microPred}^{+}\cup S_{microPred}^{-} \end{array}$$

Specifically, *S*_4_ consists of *S*^+^ (positive samples) and $S_{wei}^{-}$ (negative samples) with ratio ~1:8.8 (1,612:14,250). *S*_5_ was adopted from microPred (Batuwita and Palade, 2009), which contains 691 non-redundant human pre-miRNAs from miRBase release 12 and 8,494 pseudo hairpins.

To evaluate experimental performance on other species, we retrieved the virus pre-miRNA sequences dataset from the study of Gudyś et al. (2013). Similar to other datasets, we removed pre-miRNAs sharing more than 80% sequence similarity by the CD-HIT software. As a result, we constructed a virus dataset namely *S*_6_, which contains 232 positive samples and 232 negative samples. The benchmark dataset *S*_6_ can be formulated as:

$$\begin{array}{l} S_{6}=S_{virus}^{+}\cup S_{virus}^{-} \end{array}$$

Where the virus pre-miRNA sequences dataset *S*_6_ consists of $S_{virus}^{+}$ (positive samples) and $S_{virus}^{-}$ (negative samples), which were obtained from the study of Gudyś et al. (2013).

### Classification Algorithm and Optimization

We selected SVM to classify the samples. Specifically, the publicly available support vector machine library (LIBSVM) was applied to the benchmark data with our feature representation. The LIBSVM toolkit can be downloaded freely at http://www.csie.ntu.edu.tw/~cjlin/libsvm. We integrated this toolbox in the Matrix Laboratory (MATLAB) workspace to build the prediction system. We selected the radial basis function as the kernel function, and a grid search based on the 10-fold cross validation was used to optimize the SVM parameter γ and the penalty parameter C. C = 65,536 and γ = 10^−4^ was tuned to be the optimal parameters.

### Features Extraction

#### Primary Sequence Features Based on Mutual Information (PSFMI)

Recently, it has been shown that local continuous primary sequence characteristics are crucial for pre-miRNA prediction (Bonnet et al., 2004). As one of the important characteristics, n-grams are often used in feature mapping (Liu and Wong, 2003). Let S be a given pre-miRNA sequence (consisting of four characters: A, U, C, and G) with length L. Then the n-grams represent a continuous subsequences of length n in S with.

**Figure 2:**
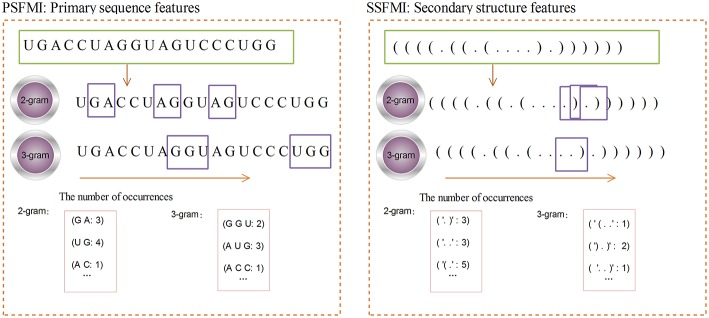
The 2-gram and 3-gram feature representation.

Figure 2 shows the calculation process for the 2-gram and 3-gram PSFMI feature representations. Any two and three consecutive bases in the pre-miRNA sequence, regardless of the order of the bases, are represented as 2- and 3-gram, respectively. For example, as shown in Figure 2, the number of bases “GA”(2-gram) is 3. The number of bases “UG”(2-gram) is 4. Similarly, 3-gram represents three consecutive bases, such as the number of bases “G G U”(3-gram) is 2.

In this study, we used entropy and mutual information (MI) to calculate the interdependence between two bases on a given pre-miRNA sequence. Specifically, we calculated the 3-gram MI and the 2-gram MI as the feature vector for a given pre-miRNA sequence. The 3-tuple MI for 3-gram is calculated as:

(1) $$\begin{array}{r} MI\left(x,y,z\right)=MI\left(x,y\right)-MI\left(x,y|z\right) \end{array}$$

where x, y, and z are three conjoint bases. Subsequently, the MI MI(x, y) and conditional MI MI(x, y|z) can be calculated as follows:

(2) MI(x,y|z)=H(x|z)−H(x|y,z)

(3) $$MI(x,y)=p(x,y)*\log(\frac{p(x,y)}{p(x)*p(y)})$$

(4) MI(x,y)=MI(y,x)

Here, *H*(*x*|*z*) and*H*(*x*|*y, z*) are calculated as follows:

(5) $$\begin{array}{r} H\left(x\right)=p\left(x\right)*\log\left(p\left(x\right)\right) \end{array}$$

(6) $$\begin{array}{r} H\left(x|z\right)=-\frac{p\left(x,z\right)}{p\left(z\right)}\log\left(\frac{p\left(x,z\right)}{p\left(z\right)}\right) \end{array}$$

(7) $$\begin{array}{r} H\left(x|y,z\right)=-\frac{p\left(x,y,z\right)}{p\left(y,z\right)}\log\left(\frac{p\left(x,y,z\right)}{p\left(y,z\right)}\right) \end{array}$$

where *p*(*x*) denotes the frequency of *x* appearing in a pre-miRNA sequence, *p*(*x, y*)denotes the frequency of *x* and *y* appearing in 2-grams and *p*(*x, y, z*) denotes the frequency of *x, y*, and *z* appearing in 3-tuples in a pre-miRNA sequence. *p*(*x*), *p*(*x, y*) and *p*(*x, y, z*) can be calculated by Equations (8)–(10):

(8) $$\begin{array}{r} p\left(x\right)=\frac{N_{x}\;+\;\varepsilon }{L} \end{array}$$

(9) $$\begin{array}{r} p\left(x,y\right)=\frac{N_{xy}\;+\;\varepsilon }{L\;-\;1} \end{array}$$

(10) $$\begin{array}{r} p\left(x,y,z\right)=\frac{N_{xyz}\;+\;\varepsilon }{L\;-\;2} \end{array}$$

(10) *N*_*x*_ is the number of occurrences of base x appearing in the pre-miRNA sequence, and L is the length of the pre-miRNA sequence. In Equation (8), ε represents a very small positive real number that does not affect the final score, which is used to avoid having 0 as the denominator.

According to the Equation (10), a given pre-miRNA sequence can be expressed as 30 mutual information values [20 3-tuples *IM (x, y, z)* and 10 2-tuples *IM (x, y)*]. In addition, we calculated the frequency of the four base classes appearing in this pre-miRNA sequence. Therefore, the pre-miRNA sequence can be expressed as 20 + 10 + 4 = 34 features, as determined using our proposed mutual information method.

#### Secondary Structure Features Based on Mutual Information (SSFMI)

**Figure 3:**
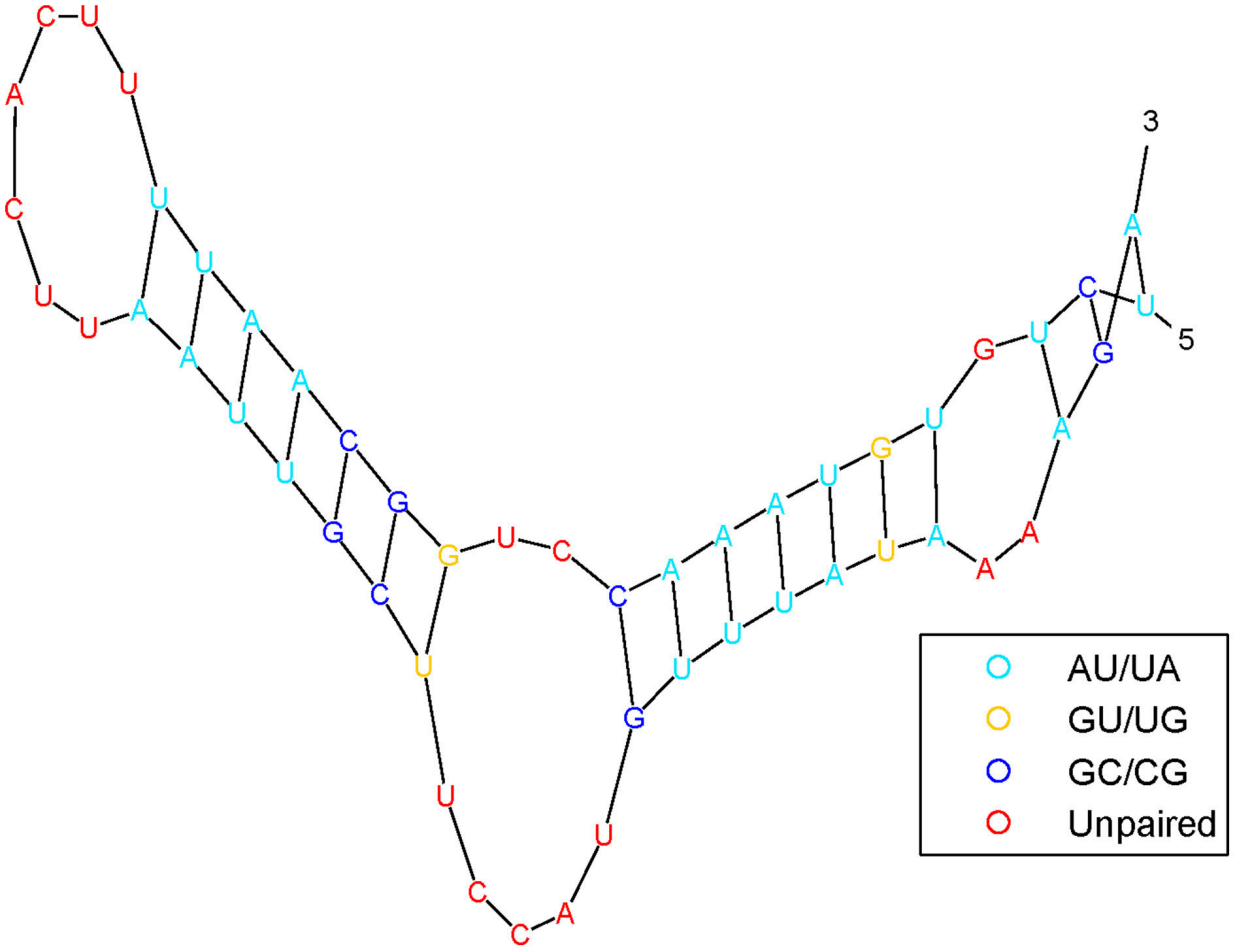
The pre-miRNA secondary structure of miRNA hsa-mir-302f.

It has been shown that the structure of pre-miRNA can provide insights into biological functions. Pre-miRNA structural information can be predicted by RNAfold (Hofacker, 2003) software from sequences and is frequently used as features by machine-learning algorithms. Figure 3 shows the pre-miRNA secondary structure of miRNA hsa-mir-302f, which was obtained using the algorithm in Mathews et al. (1999).

The pre-miRNA secondary structure is represented as a sequence of three symbols: a left parenthesis, a right parenthesis, and a point. In other words, nucleotides have only two states: paired and unpaired nucleotides, which are represented in parentheses “(” or “)” and points “.”, respectively. The open parenthesis “(” indicates that the paired nucleotides located on the 5′ end can be paired with 3′-end nucleotides, which are represented by the corresponding close parenthesis “).” The secondary structure of the pre-miRNA sequence is composed of free radicals and radical pairs A–U and C–G. To a certain extent, after such treatment, the secondary structure of the pre-miRNA sequence can be converted into a linear sequence.

A given pre-miRNA sequence S is converted to a pre-miRNA secondary structure sequence by using the RNAfold software. The length of the sequence is denoted by L, and the mutual information of the secondary structure sequence n-gram is calculated by Equations (1) and (3). The calculation process is similar to that for the PSFMI. Figure 2 shows the calculation process for the 2-gram and 3-gram SSFMI feature representations.

According to Equations (1)–(10), the pre-miRNA secondary structure sequence can be expressed as 16 mutual information values [10 3-tuples IM(x, y, z) and 6 2-tuples IM(x, y)]. Similarly, the frequencies of the three symbols that appear in the sequence of secondary structure elements were calculated. Another significant feature is the amount of base pairs in pre-miRNA sequences. For the pre-miRNA gene, given the presence of the G–U wobble pair in the hairpin loop structure (secondary structure) of the pre-miRNA, the G–U pair is considered in the base pairing.

Therefore, the secondary structure features can be expressed as 10 + 6 + 3 + 1 = 20 features, as determined using our proposed mutual information method.

In addition, studies have shown that real pre-miRNA sequences are generally more stable than randomly generated pseudo-pre-miRNAs and therefore have lower MFE. Therefore, during the process of feature extraction for pre-miRNA sequences, structural energy features are often used to characterize pre-miRNA sequences. Since the structural calculation result of RNAfold is actually provided along with the MFE value of the secondary structure of the sequence, we took this value.

In summary, we extracted a total of 55 [34 (PSFMI) + 20 (SSFMI) + 1 (MFE)] features, in which the 34-dimensional feature was obtained by applying the PSFMI method from the pre-miRNA sequence, the 20-dimensional feature was obtained by applying the SSFMI method from the pre-miRNA secondary structure, and the 1 (MFE) dimension feature is the MFE value calculated by the RNAfold software. Since the distribution of the values in each feature is non-uniform, we normalized each feature to (−1,1) using the MATLAB function mapminmax (MATLAB 2014b), and obtained the final 55-dimensional feature data set for model training.

### Measurements

In statistical prediction experiments, three cross-validation methods are often used to test the effectiveness of a prediction algorithm including independent dataset test, K-fold validation test and the Jackknife validation test. Among them, the Jackknife test is considered to be the most rigorous and objective method of verification. In the field of pre-miRNA prediction, the Jackknife tests are often used to verify the predictive performance of different algorithms. In the Jackknife test, each pre-miRNA sequence was individually selected as a test sample, and the remaining pre-miRNA sequences were used as training samples, and the test sample categories were predicted from the model trained by the training samples. Therefore, we adopted the Jackknife test in this study.

In order to comprehensively evaluate the performance of the pre-miRNA prediction method, several indicators were introduced in this paper. Receiver operating characteristic (ROC) was plotted based on specificity (Sp) and sensitivity (Sn). The areas under ROC curves (AUC) and average area under the precision-recall curve (AUPR) are both used as the evaluation metrics. The AUC provides a measure of the classifier performance; the larger the value of the AUC is, the better the performance of the classifier. However, for class imbalance problem, AUPR is more suitable than AUC, for it punishes false positive more in evaluation. In addition, Matthew correlation coefficient (MCC) was used to evaluate the prediction performance. The MCC accounts for true and false positives and negatives and are usually regarded as a balanced measure that can be used even if the classes are of different sizes. The sensitivity (SE), specificity (SP), precision (PR), accuracy (ACC), and MCC are defined as follows:

(11) $$\begin{array}{r} SE=\frac{TP}{TP+FN} \end{array}$$

(12) $$\begin{array}{r} SP=\frac{TN}{TN+FP} \end{array}$$

(13) $$\begin{array}{r} PR=\frac{TP}{TP+FP} \end{array}$$

(14) $$\begin{array}{r} F_{1}-score=2\times \frac{SE\;\times \;PR}{SE\;+\;PR} \end{array}$$

(15) $$\begin{array}{r} ACC=\frac{TP\;+\;TN}{TP\;+\;FP\;+\;TN\;+\;FN} \end{array}$$

(16) $$MCC=\frac{TP\times TN-FP\times FN}{\sqrt{\left(\text{TP+FN})(\text{TN+FP})(\text{TP+FP})(\text{TN+FN}\right)}}$$

Where TP, TN, FP, and FN denote the number of true positives, true negatives, false positives and false negatives, respectively.

## Results and Discussion

### Performance of Different Features

According to the feature extraction algorithm proposed in this paper, the corresponding 55 features (including PSFMI, SSFMI, and MFE) were extracted for each true and false pre-miRNA (positive and negative sample data) in the benchmark dataset. For the improved evaluation of these features, they were subdivided into four subsets according to the different feature types, namely, PSFMI, SSFMI, PSFMI + MFE, and SSFMI + MFE feature sets. To assess the importance of each feature subset, predictive models were constructed on the basis of the different feature subsets of the benchmark dataset. Jackknife verification was used to evaluate the performance of the predictive models.

Table 1 presents a comparison of the performances of the predictive models based on the different feature subsets and combinations thereof. As demonstrated in Table 1, the predictive model based on the feature subset SSFMI is better than that based on the feature subset PSFMI. The predictive model based on SSFMI achieves 80.21% sensitivity, 88.34% specificity, the Matthews coefficient of 0.688, and prediction accuracy of 84.27%. The predictive model based on the mutual information of pre-miRNA secondary structure is better than that based on the sequence-based mutual information. The performances of the predictive models based on the PSFMI + MFE and SSFMI + MFE feature sets are significantly improved compared with those based on the independent feature subsets (i.e., PSFMI and SSFMI feature sets). In terms of accuracy, the performance of the PSFMI + MFE model is 13.24% better than that of the PSFMI model, whereas the performance of the SSFMI + MFE based model is 1.31% better than that of the SSFMI model. The experimental results show that the combination of MFE features should be considered to increase prediction accuracy.

**Table 1:** The performance of different features on benchmark dataset (Jackknife test evaluation).

**Features**	**SE (%)**	**SP (%)**	**ACC (%)**	**MCC**
PSFMI	67.99	69.04	68.52	0.370
SSFMI	80.21	88.34	84.27	0.688
PSFMI+MFE	78.60	84.93	81.76	0.637
SSFMI+MFE	**81.95**	**89.21**	**85.58**	**0.713**

*The best values are shown in boldface*.

**Figure 4:**
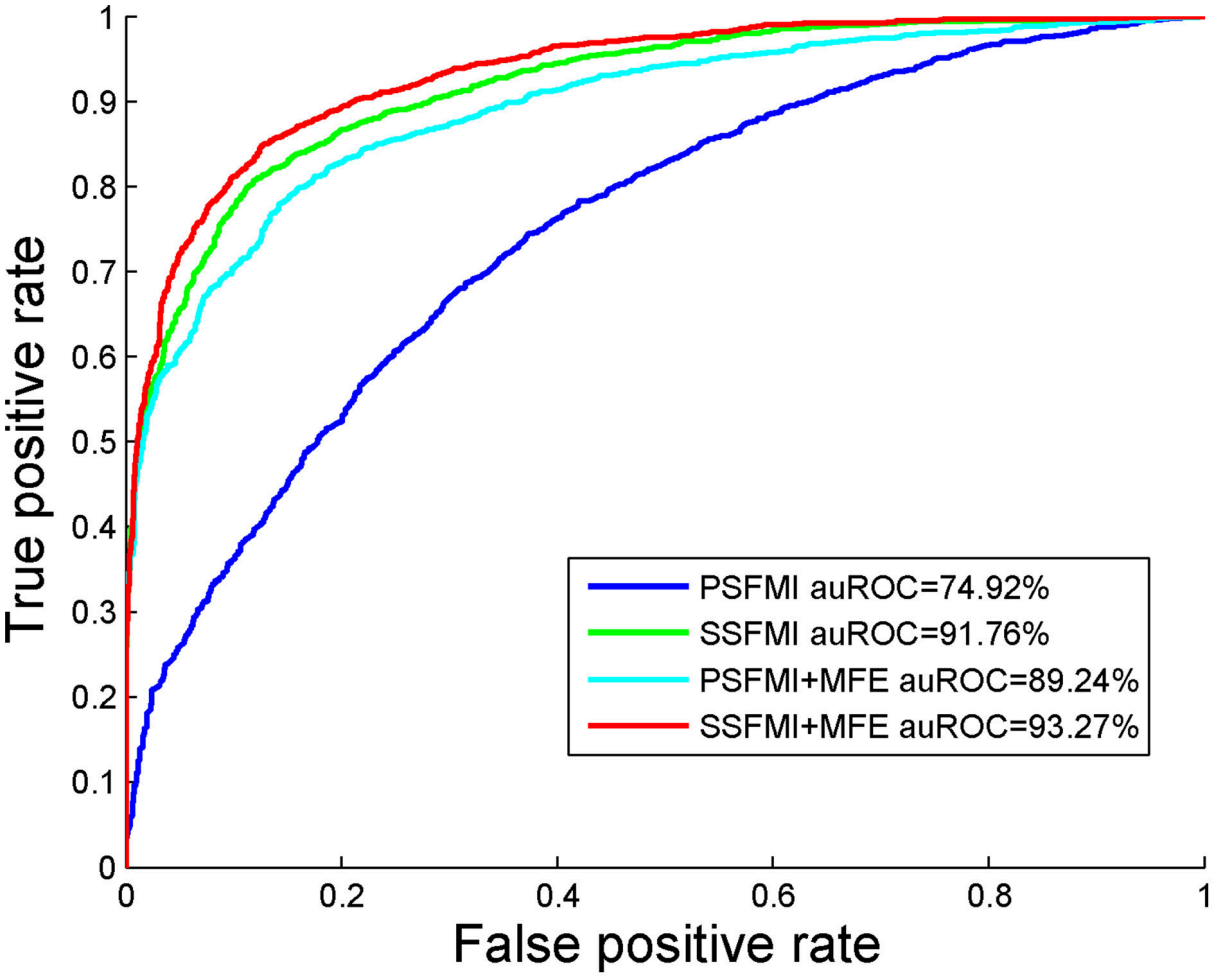
The AUROC comparison of four feature combinations through the Jackknife cross-validation.

We also compare the AUROC of four feature combinations obtained by Jackknife cross-validation on benchmark dataset *S*_1_, shown in Figure 4. We can draw the same conclusion that the prediction model based on feature subset SSFMI is better than the prediction model based on feature subset PSFMI, and the combination of MFE features can improve the accuracy of prediction.

### Feature Importance Analysis

To explore the extent to which the features in the feature set affect the classification, we analyzed the importance of each feature in the feature set. To quantitatively measure the importance of each feature, we introduced the metric information gain (IG) (Deng et al., 2011; Uǧuz, 2011). IG scores are widely used in the analysis of feature importance of biological sequences (Wei et al., 2014, 2017; Chen et al., 2016). The higher the value of IG, the more important the feature is for the classifier. Table 2 presents the IG scores of 55 features. As shown in Table 2, although the 4 highest IG values all belong to PSFMIs, the 10 lowest IG values also belong to PSFMIs, indicating that the IG values of the PSFMIs are unevenly distributed and have large differences. The average IG value of PSFMI features is 0.5761, whereas the average IG value of SSFMI is 0.7489, further confirming that the secondary structure characteristics of pre-miRNA have a greater influence on the classification results than the primary sequence characteristics. The experimental findings are also consistent with the feature importance analysis.

**Table 2:** Importance of the relatively specific features in the proposed features set.

**Rank**	**Features**	**IG**
1	PSFMI_1	1.0000
2	PSFMI_2	1.0000
3	PSFMI_3	0.9981
4	PSFMI_4	0.9975
5	SSFMI_1	0.9963
6	PSFMI_5	0.9963
7	PSFMI_6	0.9933
8	SSFMI_2	0.9890
9	SSFMI_3	0.9772
10	PSFMI_7	0.9750
11	PSFMI_8	0.9739
12	PSFMI_9	0.9722
13	PSFMI_10	0.9717
14	SSFMI_4	0.9680
15	PSFMI_11	0.9625
16	SSFMI_5	0.9608
17	PSFMI_12	0.9423
18	PSFMI_13	0.9143
19	SSFMI_6	0.8940
20	SSFMI_7	0.8936
21	PSFMI_14	0.8916
22	SSFMI_8	0.8909
23	SSFMI_9	0.8870
24	SSFMI_10	0.8787
25	SSFMI_11	0.8624
26	PSFMI_15	0.8429
27	SSFMI_12	0.8387
28	SSFMI_13	0.8364
29	SSFMI_14	0.8282
30	PSFMI_16	0.7897
31	PSFMI_17	0.7859
32	PSFMI_18	0.7851
33	PSFMI_19	0.7386
34	PSFMI_20	0.6681
35	SSFMI_15	0.6008
36	SSFMI_16	0.6008
37	SSFMI_17	0.6008
38	SSFMI_19	0.5995
39	MFE	0.4575
40	PSFMI_21	0.3508
41	PSFMI_22	0.3504
42	PSFMI_23	0.3351
43	PSFMI_24	0.3218
44	SSFMI_19	0.2647
45	SSFMI_20	0.1044
46	PSFMI_25	0.0058
47	PSFMI_26	0.0057
48	PSFMI_27	0.0052
49	PSFMI_28	0.0032
50	PSFMI_29	0.0025
51	PSFMI_30	0.0025
52	PSFMI_31	0.0023
53	PSFMI_32	0.0009
54	PSFMI_33	0.0005
55	PSFMI_34	0.0003

### Effect of Different Kernel Functions

To justify different kernel functions of SVM for our algorithm, we ran another set of experiments on the benchmark dataset using Jackknife test evaluation. Several kernel functions were tested in the experiments: SVM with linear kernel, SVM with polynomial kernel, SVM with Radial Basis Function (RBF) kernel and SVM with sigmoid kernel. The results achieved in these experiments are shown in Table 3. We could see the ACC, MCC, and AUC of the SVM classifier with RBF kernel outperformed all other classifiers. Therefore, in this study, we choose the SVM classifier of the RBF kernel.

**Table 3:** Comparison of performance of different kernel functions on the benchmark dataset *S*_1_ (Jackknife test evaluation).

**Methods**	**SE (%)**	**SP (%)**	**ACC (%)**	**MCC**	**AUC (%)**
SVM (linear kernel)	88.83	92.12	90.48	0.810	96.20
SVM (polynomial kernel)	84.86	85.86	85.36	0.707	93.04
SVM (rbf kernel)	88.59	**92.62**	**90.60**	**0.813**	**96.54**
SVM (sigmoid kernel)	**88.96**	91.94	90.45	0.809	96.26

*The best values are shown in boldface*.

### Performance on Balanced Dataset

We compared the ACC, SE, SP, MCC, and AUC achieved on the benchmark dataset *S*_1_ and ***S***_**2**_ by our predictor with the following methods: iMiRNA-SSF (Chen et al., 2016), miRNAPre (Wei et al., 2014), Triplet-SVM (Xue et al., 2005), iMcRNA-PseSSC (Liu et al., 2015b), and iMiRNA-PseDPC (Liu et al., 2016), and A brief introduction to these methods is shown in Table 4. As can be seen from Table 4, both the iMcRNA-PseSSC (Liu et al., 2015b) and iMiRNA-PseDPC (Liu et al., 2016) methods require parameters, and the iMiRNA-PseDPC (Liu et al., 2016) method features the largest dimension.

**Table 4:** A brief introduction to the state-of-the-art predictors.

**Methods**	**Classifier**	**Dimensions**	**Parameters**
Triplet-SVM	SVM	32	No parameter
miRNAPre	SVM	98	No parameter
iMiRNA-SSF	SVM	98	No parameter
iMcRNA-PseSSC	SVM	113	*n* = 2, λ = 13, ω = 0.5^*a*^
iMiRNA-PseDPC	SVM	725	*d* = 7, λ = 15, ω = 1^*a*^
Our method	SVM	55	No parameter

a *ACC's best parameter settings*.

The performance of different methods on the benchmark datasets *S*_1_ and *S*_2_ via the jackknife test, as showed in Tables 5, 6, respectively. For a fair comparison, the performances of these methods were taken from other studies with best tuned parameters (Liu et al., 2015b, 2016). Table 5 shows that our method significantly outperforms previous methods in all evaluation metrics used. Among the evaluated methods, our method achieves the best predictive performance on four metrics: AUC (96.54%), ACC (90.60%), MCC (0.813), and SP (92.62%). The respective ACC and MCC of our method are 1.51% and 0.051 higher than those of the previously known best-performing predictor iMiRNA-SSF (Chen et al., 2016) (ACC = 88.09% and MCC = 0.762). The AUC of our method is 1.57% higher than those of the previously known best-performing predictor iMiRNA-PseDPC (Liu et al., 2016) (AUC = 94.97%). In addition, We have incorporated the new negative samples from Wei's study (Wei et al., 2014) to construct a new benchmark dataset *S*_2_, and compared the prediction performance of our method together with 5 other popular methods using the Jackknife test (see Table 6). As can be seen, our method achieves the best predictive performance on 4 (out of 5) metrics including AUC (95.04%), ACC (88.00%), MCC (0.760), and specificity (88.71%), and is slightly worse than iMiRNA-PseDPC in sensitivity.

**Table 5:** Results of the proposed method and state-of-the-art predictors on benchmark dataset *S*_1_ (Jackknife test evaluation).

**Methods**	**SE (%)**	**SP (%)**	**ACC (%)**	**MCC**	**AUC (%)**
iMiRNA-SSF	**89.27**	86.91	88.09	0.762	94.64
Triplet-SVM	82.44	85.24	83.84	0.677	91.97
miRNAPre	84.24	87.90	86.07	0.722	93.49
iMcRNA-PseSSC	84.55	86.41	85.48	0.710	93.22
iMiRNA-PseDPC	86.72	89.21	87.97	0.760	94.97
Our method	88.59	**92.62**	**90.60**	**0.813**	**96.54**

*The best values are shown in boldface*.

**Table 6:** Results of the proposed method and state-of-the-art predictors on benchmark dataset *S*_2_ (Jackknife test evaluation).

**Methods**	**SE (%)**	**SP (%)**	**ACC (%)**	**MCC**	**AUC (%)**
iMiRNA-SSF	84.49	85.86	85.17	0.704	92.03
Triplet-SVM	82.07	84.12	83.10	0.662	90.86
miRNApre	84.80	86.79	85.79	0.716	92.81
iMcRNA-PseSSC	80.02	82.75	81.39	0.628	89.71
iMiRNA-PseDPC	**87.66**	87.16	87.41	0.748	94.79
Our method	87.28	**88.71**	**88.00**	**0.760**	**95.04**

*The best values are shown in boldface*.

To further compare the performance of our method with other methods on independent testing, we chose the *S*_1_ dataset as the training set and the *S*_3_ dataset as the test set. Table 7 shows that our method outperforms all other methods in the independent test with an ACC of 70.45% and MCC of 0.412. The iMiRNA-PseDPC (Liu et al., 2016) method has an AUC value of 81.69%, which is the best AUC value in all methods. The AUC of our method (AUC = 75.54%) is comparable to the AUC of the iMcRNA-PseSSC (Liu et al., 2015b) method (AUC = 75.81%). The dimensions of iMiRNA-PseDPC are as high as 725 dimensions, far exceeding the 55-dimensional of our method, and the time overhead of our method is less than iMiRNA-PseDPC.

**Table 7:** Comparing the proposed method with other state-of-the-art predictors on an independent dataset *S*_3_.

**Methods**	**SE (%)**	**SP (%)**	**ACC (%)**	**MCC**	**AUC (%)**
miRNApre	80.68	48.86	64.77	0.312	72.02
iMiRNA-PseDPC	**84.09**	47.73	65.91	0.342	**81.69**
iMcRNA-PseSSC	80.68	56.82	68.75	0.386	75.81
Triplet-SVM	80.68	46.59	63.64	0.290	68.92
Our method	76.14	**64.77**	**70.45**	**0.412**	75.54

*The best values are shown in boldface*.

### Performance on Imbalanced Dataset

**Figure 5:**
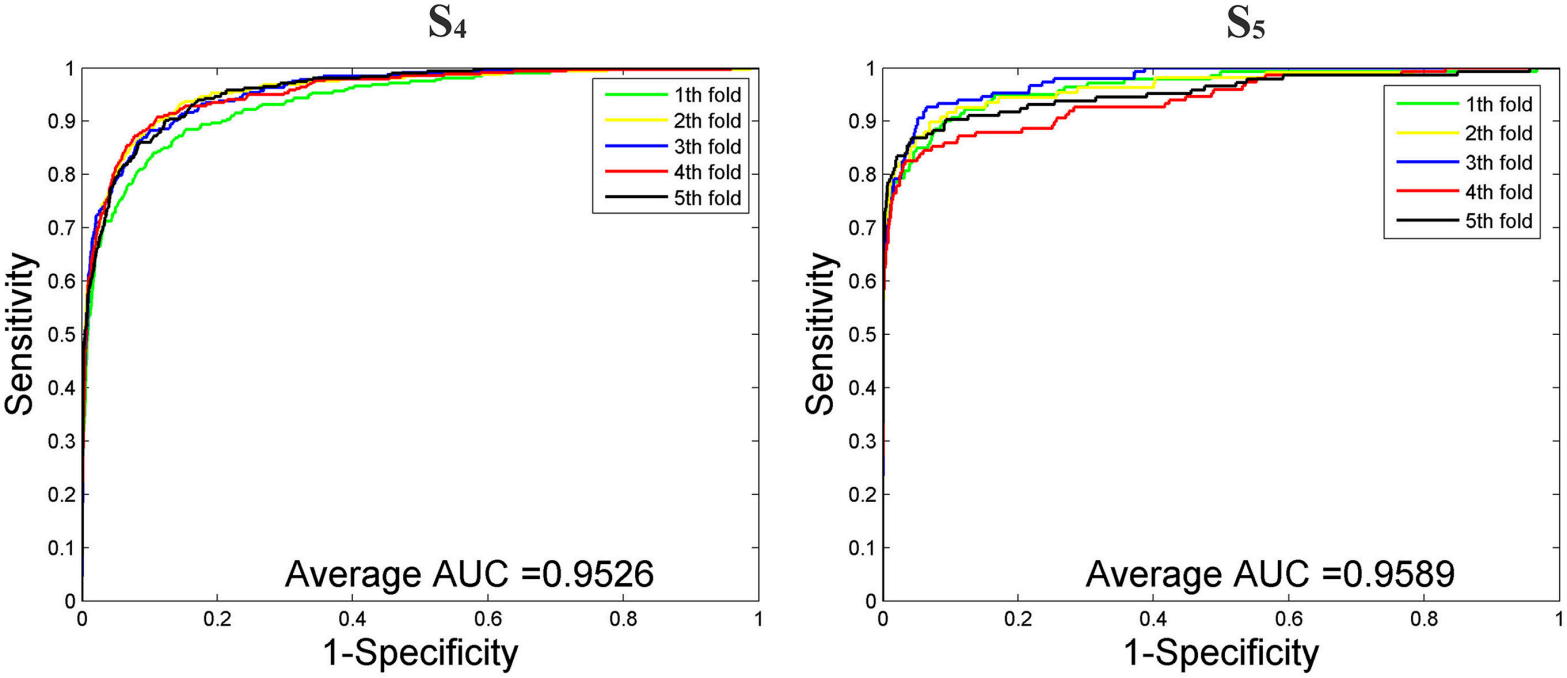
The AUROC curves of our method on the imbalanced benchmark dataset *S*_4_ and *S*_5_ via 5-fold cross validation.

**Figure 6:**
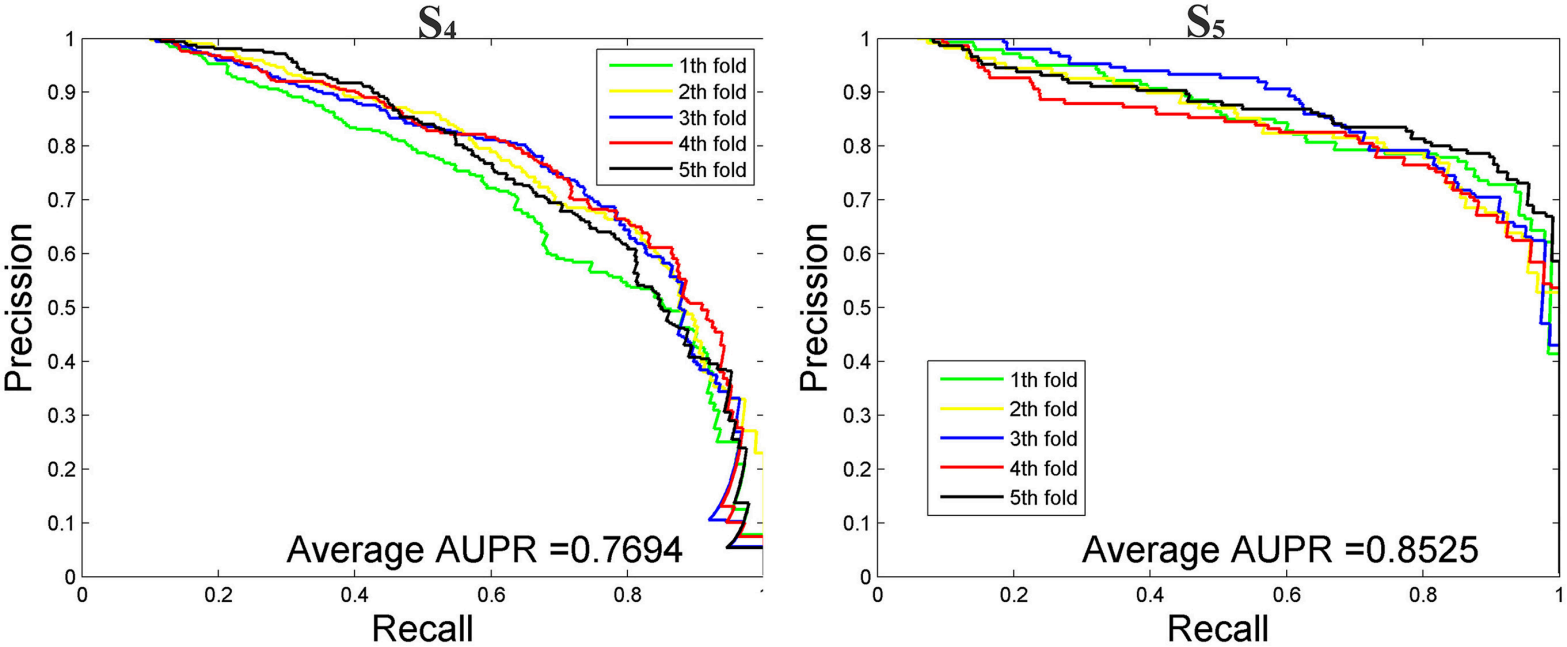
The AUPR curves of our method on the imbalanced benchmark dataset *S*_4_ and *S*_5_ via 5-fold cross validation.

We then tested our method on *S*_4_ and *S*_5_ together with the other 4 State-of-the-Arts methods including miRNAPre (Wei et al., 2014), Triplet-SVM (Xue et al., 2005), iMcRNA-PseSSC (Liu et al., 2015b), and iMiRNA-PseDPC (Liu et al., 2016). The performance was evaluated using the 5-fold cross validation and the results were summarized in Table 8. As can be seen, our method performed the best for all 3 evaluation metrics including AUC (0.9589), *F*_1_*score* (0.7813), and AUPR (0.8525), respectively on the dataset *S*_5_. As for the dataset *S*_4_, our method ranks the first on *F*_1_*score* (with a value 0.7084) and second on AUC and AUPR. For a better view, we also plotted the AUC curves and AUPR curves of our method on *S*_4_ and *S*_5_ for all 5-folds, respectively in Figures 5, 6.

**Table 8:** Five-fold cross-validation prediction performance of the proposed method and 4 state-of-the-art predictors on imbalanced benchmark dataset *S*_**4**_ and *S*_**5**_.

**Methods**	**AUC**	***S*_4_*F*_1 score_**	**AUPR**	**AUC**	***S*_5_*F*_1 score_**	**AUPR**
iMcRNA-PseSSC	0.9103	0.5707	0.6743	0.9333	0.7157	0.7628
iMiRNA-PseDPC	0.9333	0.6404	0.7317	0.9534	0.7708	0.8259
Triplet-SVM	0.8905	0.5207	0.6364	0.9357	0.7182	0.7806
miRNApre	**0.9554**	0.7029	**0.7976**	0.9454	0.7447	0.8140
Our method	0.9526	**0.7084**	0.7694	**0.9589**	**0.7813**	**0.8525**

*The best values are shown in boldface*.

### Performance on Other Species

We then compared our method with 4 state-of-the-arts methods on the benchmark dataset *S*_6_ through the jackknife test. Table 9 shows that our method outperforms all other methods in the independent test with an ACC of 92.59%, MCC of 0.852, and AUC of 98.07%. The experimental results show that our method also has good performance on other species.

**Table 9:** Comparing the proposed method and state-of-the-art predictors on the benchmark dataset *S*_6_ (using the Jackknife test).

**Methods**	**SE (%)**	**SP (%)**	**ACC (%)**	**MCC**	**AUC (%)**
miRNApre	92.59	88.43	90.51	0.811	97.11
Triplet-SVM	89.35	88.43	88.89	0.778	95.85
iMiRNA-PseDPC	91.67	91.67	91.67	0.833	97.41
iMcRNA-PseSSC	89.81	87.96	88.89	0.778	95.55
Our method	**93.06**	**92.13**	**92.59**	**0.852**	**98.07**

*The best values are shown in boldface*.

### Case Study

Sometimes, the lower version of miRBase database (e.g., miRBase 20) may contain some false-positive pre-miRNAs, which will be excluded in a later version (e.g., miRBase 22). Usually, they are saved in the file “miRNA.dead.” Obviously, if we used miRBase 20 as a bench-mark data, a good method should predict the false-positive pre-miRNAs to be negative (i.e., not to be pre-miRNAs). Fortunately, it is the case for our method and we listed the 8 predicted false-positive pre-miRNAs in Table 10, in which the column names “ID” and “Accession” indicate the Id number and the Accession number of the pre-miRNA sequences in miRbase 22, respectively.

**Table 10:** False-positive pre-miRNAs predicted to be negative by our method.

**ID**	**Accession**
hsa-mir-566	MI0003572
hsa-mir-3607	MI0015997
hsa-mir-3656	MI0016056
hsa-mir-4417	MI0016753
hsa-mir-4459	MI0016805
hsa-mir-4792	MI0017439
hsa-mir-6723	MI0022558
hsa-mir-7641-1	MI0024975

### Running Time

In this study, we used the SVM model to predict pre-miRNAs. The time complexity of training our SVM model is $O\left(N_{S}^{3}+N_{S}^{2}.l+N_{S}.\text{d}.l\right)$ (Burges, 1998). Where *l* is the number of training points, *N*_*S*_ is the number of support vectors (SVs), and d is the dimension of the input data.

To further evaluate the performance of our method and other competitors, we tested the running time on *S*_6_ datasets on the same platform. The experiments were carried out on a computer with Intel(R) Xeon(R) CPU E5-2650 0@2.00GHz 2.00GHz, 16GB memory and Windows OS. Detailed results of running time were shown in Table 11. Our method achieves the better performance of running time, and obtains a good performance of accuracy.

**Table 11:** The running time (in seconds) of different methods on benchmark dataset *S*_6_ using the Jackknife test, where C and γ represent the penalty coefficient of the SVM model and the parameters of the RBF function, respectively.

**Methods**	**γ**	**C**	**Time**	**ACC (%)**	**AUC (%)**
miRNApre	0.25	16	133.159	90.51	97.11
Triplet-SVM	0.0156	2	21.689	88.89	95.85
iMiRNA-PseDPC	0.25	16	543.803	91.67	97.41
iMcRNA-PseSSC	0.0039	39	50.459	88.89	95.55
Our method	0.0156	16	19.712	92.59	98.07

## Conclusions

Pre-miRNA prediction is one of the hot topics in the field of miRNA research (Yue et al., 2014; Cheng et al., 2015; Liao et al., 2015a; Luo et al., 2017, 2018; Peng et al., 2017, 2018; Xiao et al., 2017; Fu et al., 2018). In recent years, machine learning-based miRNA precursor prediction methods have made great progress. Most of the existing prediction methods are based on the global feature extraction feature of the sequence, ignoring the influence of the sequence base characters, and the pre-miRNA structure information does not consider the local characteristics. For this reason, this paper performs mutual information calculation on the pre-miRNA sequence and the secondary structure, respectively, to extract the pre-miRNA sequence and the local features of the secondary structure. Then, the extracted features are input to a support vector machine classifier for prediction.

Finally, the experimental results show that: compared with the existing methods, the proposed method improves the sensitivity and specificity of pre-miRNA prediction. In addition, since the feature space of our method is only 55, less than that of most state-of-the-art methods, our feature construction is also efficient when plugging into canonical classification methods such as SVM. In summary, our method can extract effective features of pre-miRNAs and predicts reliable candidate pre-miRNAs for further experimental validation.

## Author Contributions

XF, JY, YC, BL, and LC conceived the concept of the work. XF, WZ, and LP performed the experiments. XF and JY wrote the paper.

### Conflict of Interest Statement

The authors declare that the research was conducted in the absence of any commercial or financial relationships that could be construed as a potential conflict of interest.
